# Lower serum testosterone is associated with increased likelihood of arthritis

**DOI:** 10.1038/s41598-023-46424-1

**Published:** 2023-11-07

**Authors:** Lulu Cheng, Siyu Wang

**Affiliations:** 1 College of Acupuncture-Moxibustion and Tuina, Anhui University of Chinese Medicine, Hefei, 230012 China; 2 Graduate School, Wuhan Sports University, Wuhan, 430079 China

**Keywords:** Diseases, Medical research, Rheumatology

## Abstract

Studies have suggested that serum testosterone levels may be strongly correlated with the pathogenesis of arthritis. Therefore, the aim of this study was to assess the relationship between serum testosterone levels and arthritis in US adults using the National Health and Nutrition Examination Survey (NHANES). We used the database from NHANES, 2013–2016 to perform a cross-sectional study. This study investigated the relationship between serum testosterone and arthritis using multivariate logistic regression models and also used smoothed curve fitting and generalized additivity models. A total of 10,439 adults were included in this analysis. A significant negative association between serum testosterone and arthritis was found in a linear regression analysis. The study showed that the arthritis group had lower testosterone levels than the non-arthritis group. The univariate multivariate analyses of Q4, using Q1 as a reference, all showed a significantly lower risk of developing arthritis. In subgroup analyses, the negative correlation between serum testosterone levels and arthritis was more significant in women and those with a body mass index (BMI) ≥ 30 kg/m^2^. After controlling for various variables, we found a significant association between serum testosterone and arthritis in this analysis. Further study of the relationship between testosterone and arthritis is necessary to clarify the specific mechanism of serum testosterone action on arthritis.

## Introduction

Arthritis is a disabling degenerative joint disease affecting the hyaline articular cartilage as well as the surrounding tissues and subchondral bone. Its etiology is unknown, and it is a multifactorial, total joint disease^1^. There are many types of arthritis, the best known diseases being osteoarthritis (OA), rheumatoid arthritis (RA), and psoriatic arthritis (PsA). OA is a degenerative disease, and PsA and RA are autoimmune diseases, and although these diseases belong to different types of arthritis, they share a number of common risk factors and causative features that are closely related to immune abnormalities. Among them, Knee osteoarthritis(KOA) has been ranked as the 11th most disabling disease in the world along with other arthritis^2^, Its risk of disability is as high as 40% in older men and 47% in older women^3^, the incidence of OA is similar in men and women up to the age of 50 years, after which the incidence increases significantly in women^4,5^, suggesting that hormonal factors have an impact on the progression and development of the disease. Researchers have addressed this issue in depth^6,7^: serum 17β estradiol and testosterone play an essential role in the development of KOA. It has been proven that estrogen is significantly associated with the development of OA. Despite awareness of testosterone's effects on other musculoskeletal tissues, little information is known about how it impacts joint cartilage or OA^8^. The relationship between testosterone and OA has been debated in clinical and basic research^9,10^. Research has shown that in a group of healthy middle-aged men without symptoms or risk factors for KOA, serum-free testosterone levels were associated with the rate of tibial cartilage loss leading to the development of arthritis at 2 years^11^. It has been shown that androgens can trigger several genomic and non-genomic pathways, and that the strongest evidence that cellular effects can be triggered through non-genomic signaling is the rapid increase in intracellular calcium concentration^12,13^. Koelling et al.^14^ found that physiological doses of testosterone had a positive effect on cartilage formation in men with advanced OA, and that testosterone replacement therapy given in synovial fluid stimulated the regenerative potential of articular cartilage tissue in patients. Colvard suggested that androgens are directly involved in the growth and development of osteoblasts, and in vitro cultured osteoblasts, mRNA transcribed from the androgen receptor (AR) gene could be directly detected and AR protein could be expressed, proving that androgens have a direct effect on osteoblasts^15^. Yan et al.^10^ found little evidence of a sex-specific association between serum testosterone levels and OA. However, whether serum testosterone levels in arthritis patients show correlated changes with the development of arthritis has not yet been clearly elucidated.

The National Health and Nutrition Examination Survey (NHANES) is a cross-sectional survey database that collects information on the health and nutrition of the US. household population. The database sample was selected using stratified multistage sampling to obtain a representative sample of U.S. residents. The data collection method consists of a household interview and a health examination. The interview included demographic and socioeconomic information as well as diet and health-related data, while the physical examination part collected data from physical examination records and laboratory tests. Therefore, we conducted a cross-sectional study based on the NHANES database (2013–2016) data to assess the potential association between serum testosterone levels and arthritis with the aim of providing new ideas for the treatment and prevention of arthritis.

## Materials and methods

### Study population

**Figure 1:**
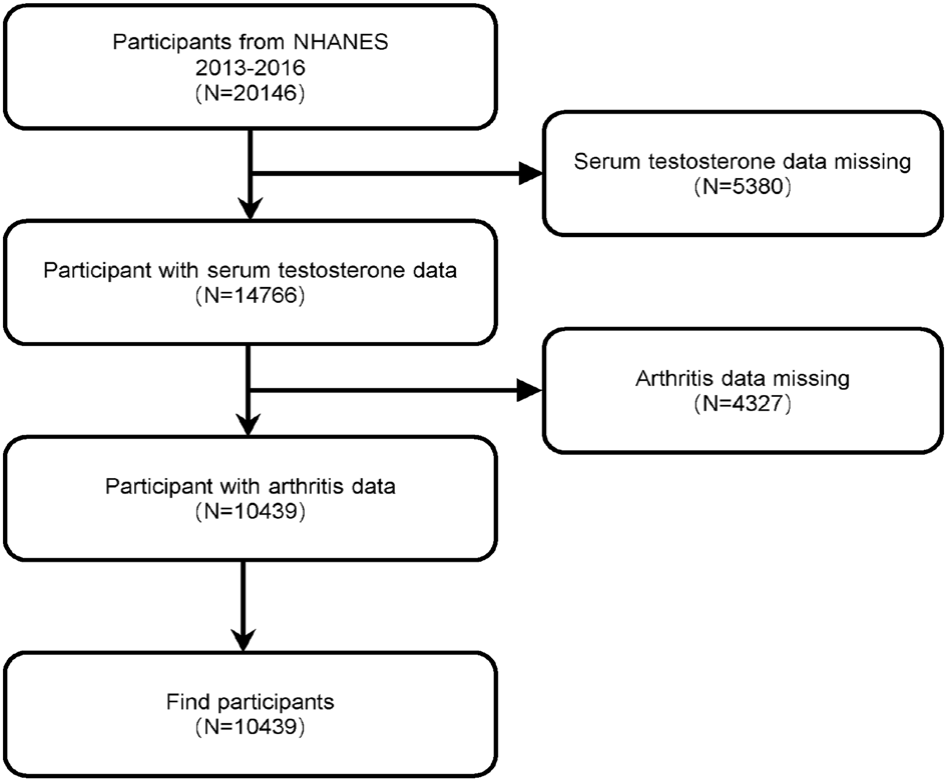
Flowchart of participant selection. NHANES, National Health and Nutrition Examination Survey.

The NHANES database is an ongoing U.S. national population-based nutrition and health survey. It uses complex, multi-stage, and probability sampling techniques rather than a simple random sample based on the U.S. population. More information about the data can be found at (https://www.cdc.gov/nchs/nhanes/index.htm). The website details the continuous design of the NHANES survey, that all study procedures were authorized by the National Center for Health Statistics Ethics Review Board prior to data collection, and that all participants signed informed consent forms. The survey utilized household questionnaires, telephone interviews, and examinations conducted by healthcare professionals and trained personnel to collect data. In the investigation, we removed 5380 participants with missing testosterone values and 4327 people with missing arthritis data from the 20,146 eligible individuals. This study included a total of 10,439 people mainly aged 20 years and older. Figure 1 depicts the sample selection.

### Study variables

The Division of Environmental Health Laboratory Sciences of the National Center for Environmental Health received, processed, and shipped the serum samples for analysis. The CDC-developed isotope dilution liquid chromatography tandem mass spectrometry (ID-LC-MS/MS) method was used to determine the concentration of TT. In-depth instructions for quality assurance and quality control were covered in the NHANES Laboratory/Medical Technologists. For more information on NHANES laboratory testosterone assays, please visit: https://wwwn.cdc.gov/Nchs/Nhanes/2013-2014/TST_H.htm.

The “Medical conditions” section of the American National Health Interview survey serves as the foundation for the NHANES Medical Conditions Questionnaire, which also probes at and collects data on arthritis status. Participants were asked if they had ever been told by a physician or other health professional that they had arthritis. If yes, they were asked to classify the arthritis diagnosis as OA or degenerative arthritis, Rheumatoid arthritis, and other. Physician-diagnosed OA in self-report is the predominant case definition used in epidemiology^16^, and in previous studies reported an agreement of 81% between self-reported and clinically confirmed OA^17^.

The covariates include demographic data, test results, lab results, and survey results. Age, sex, race, level of education, marital status, and the income-to-poverty ratio were all included in the demographic data. Waistline and body mass index (BMI) were examined data. Estradiol (pg/mL) and sex hormone binding globulin (SHBG, nmol/L) were measured in the laboratory. Finally, questionnaire results included information on alcohol use (ever or never), Smoked ≥ 100 cigarettes in life, hypertension, diabetes, and cardiovascular health. The NHANES Survey Methods and Analysis Guide (https://www.cdc.gov/nchs/nhanes/) comprehensive information on variable collection techniques.

### Statistical analysis

To examine the association between serum testosterone and arthritis, multivariate logistic regression analysis between serum testosterone and arthritis was used to construct multivariate tests, using three models with no covariates in model 1. Model 2 was adjusted for age, sex, and race. Model 3 is adjusted according to age, sex, race, education level, marital status, BMI, alcohol status, smoking status, hypertension, diabetes, cardiovascular disease (CVD), estradiol, SHBG, and Income to poverty ratio. Ratio (OR) and 95% confidence interval (CI) were used in the models to assess serum testosterone and arthritis. Multivariate tests were constructed using three models by controlling for variables and smoothing the fitted curves. The corr cardiovascular disease elation between testosterone and arthritis was investigated using a threshold effects analysis model. Finally, the same statistical analysis procedures as previously described were used for subgroups based on sex, BMI, age, hypertension, and hyperglycemia. Statistical analyses were performed using R studio (version 4.2.2) and EmpowerStats (version 4.1). *P*-value < 0.05 was determined to be significant and in addition, we used a weighting strategy to reduce large fluctuations in the dataset.

### Ethics approval and consent to participate

This is an observational study. The Research Ethics Committee of the institute the senior authors are affiliated has confirmed that no ethical approval is required. Data collection agencies obtained informed consent from all participants included in the study.

## Results

### Baseline characteristics

A total of 10,439 participants were enrolled in this study, of whom 48.11% were male and the mean age was 47.25 ± 16.80. The mean plasma testosterone level was 214.97 ± 234.70, of whom 26.97% had arthritis. The clinical characteristics of the participants according to arthritis as a column stratification variable are shown in Table 1. The presence or absence of arthritis was associated with age, sex, race, marriage, BMI, waist circumference, education, smoking status, estradiol, SHBG, hypertension, diabetes mellitus, and cardiovascular disease. It was statistically significant (*P* < 0.05). Participants with arthritis were more likely to be female if they were older compared to the non-arthritis group. Among these participants the prevalence was higher if they had smoking, waist circumference, BMI ≥ 30 kg/m^2^, hypertension, diabetes, and cardiovascular disease.

**Table 1:** Weighted characteristics of the study population based on arthritis.

	Non-arthritis (N = 7705)	Arthritis (N = 2734)	*P*-value
Age (years)	43.36 ± 15.94	59.90 ± 13.60	< 0.0001
Sex (%)			< 0.0001
Male	51.73	38.31	
Female	48.27	61.69	
Race (%)			< 0.0001
Mexican American	10.58	4.77	
Other Hispanic	6.76	3.52	
Non-Hispanic white	61.64	76.38	
Non-Hispanic black	11.40	9.55	
Other race	9.61	5.78	
Education level (%)			< 0.0001
Less than 9th grade	5.18	5.08	
9th-11th grade	9.01	10.58	
High school graduate/GED or equivalent	20.93	22.03	
Some college or AA degree	32.20	34.49	
College graduate or above	32.66	27.77	
Not recorded	0.03	0.05	
Marital, N (%)			< 0.0001
Married/living with partner	64.45	62.12	
Separated/Divorced/Widowed	14.28	29.24	
Never married	21.27	8.64	
Body mass index (kg/m^2^)			< 0.0001
< 25	32.03	18.77	
25–29.9	33.38	30.23	
≥ 30	34.59	55.24	
Alcohol status			0.1095
Yes	76.88	75.34	
No	23.12	24.66	
Smoked ≥ 100 cigarettes in life (%)			< 0.0001
Yes	40.30	52.33	
No	59.70	47.67	
Hypertension (%)			< 0.0001
Yes	25.58	55.57	
No	74.42	44.43	
Diabtes (%)			< 0.0001
Yes	7.78	18.24	
No	92.22	81.76	
CVD (%)			
Yes	5.01	18.56	
No	94.99	81.44	
Which type of arthritis was it? (%)			
Osteoarthritis or degenerative arthritis	–	52.00	
Rheumatoid arthritis	–	14.34	
Psoriatic arthritis	–	1.17	
Other	–	9.98	
Do not know or refused	–	22.51	
Waist circumference (cm)	98.05 ± 16.17	105.68 ± 16.46	< 0.0001
Testosterone (ng/dL)	233.91 ± 239.14	163.67 ± 213.96	< 0.0001
Estradiol (pg/mL)	70.24 ± 466.21	37.08 ± 303.18	0.0005
SHBG (nmol/L)	63.69 ± 63.23	64.96 ± 43.34	0.3426
Income to poverty ratio	2.97 ± 1.66	2.96 ± 1.63	0.7519

### Association between serum testosterone and arthritis

**Figure 2:**
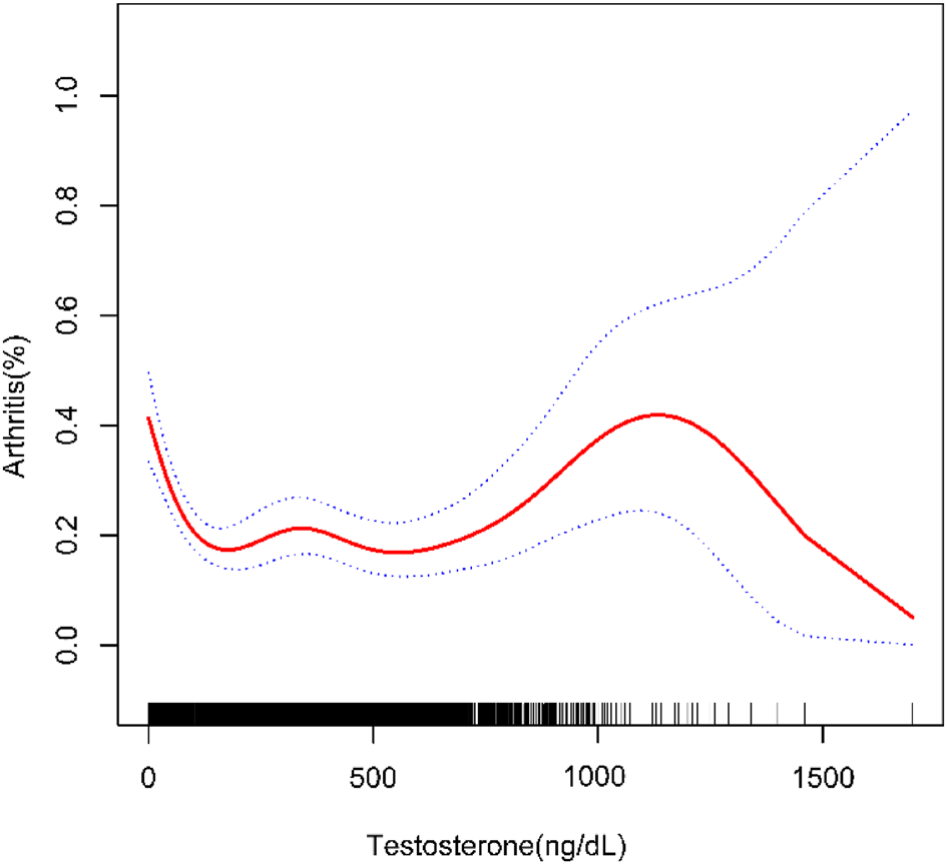
The association between serum testosterone and arthritis. The solid red line represents the smooth curve fit between variables. Blue bands represent the 95% confidence interval from the fit.

Because the effect value is not apparent, serum testosterone/100 is used to amplify the effect value by 100 times. Table 2 showed the results of the multivariable regression analysis between serum testosterone/100 and arthritis. This association was significant both in model 1 [0.87 (0.85, 0.89)] and model 2 [0.93(0.90, 0.97)]. However, in model 3, the negative association between serum testosterone and arthritis became insignificant [1.02 (0.97, 1.07)]. Sensitivity analysis using testosterone quartiles resulted in ORs of 1.00, 0.85 (0.72, 1.00), 0.53 (0.35, 0.79), and 0.49 (0.31, 0.76) for Q1, Q2, Q3, and Q4 in model 3, respectively. Participants in the highest quartile group had a 51% reduced risk of developing arthritis compared to those in the lowest quartile of testosterone levels (*p* for trend < 0.05). After adjusting for all covariates, the smoothed curve fit demonstrated the nonlinear relationship between arthritis with serum testosterone (Fig. 2).

**Table 2:** The association between serum testosterone and arthritis.

	Model1 [OR (95% CI)]	Model2 [OR (95% CI)]	Model3 [OR (95% CI)]
Serum testosterone/100 (continuous)	0.87 (0.85, 0.89)	0.93 (0.90, 0.97)	1.02 (0.97, 1.07)
Q1 (0.52–18.77)	Reference	Reference	Reference
Q2 (18.8–55.1)	0.57 (0.51, 0.64)	0.86 (0.75, 0.98)	0.85 (0.72, 1.00)
Q3 (55.2–377.98)	0.53 (0.47, 0.60)	0.66 (0.48, 0.90)	0.53 (0.35, 0.79)
Q4 (378–2000)	0.35 (0.31, 0.40)	0.50 (0.35, 0.70)	0.49 (0.31, 0.76)
*P* for trend	< 0.0001	< 0.0001	0.0327

Model 1, no covariates were adjusted.

Model 2, age, sex, and race were adjusted.

Model 3, age, sex, race, education level, marital status, BMI, alcohol status, smoking status, hypertension, diabetes, CVD, estradiol, SHBG, income to poverty ratio, were adjusted. 95% CI, 95% confidence interval; OR, odds ratio.

### Subgroup analysis

**Figure 3:**
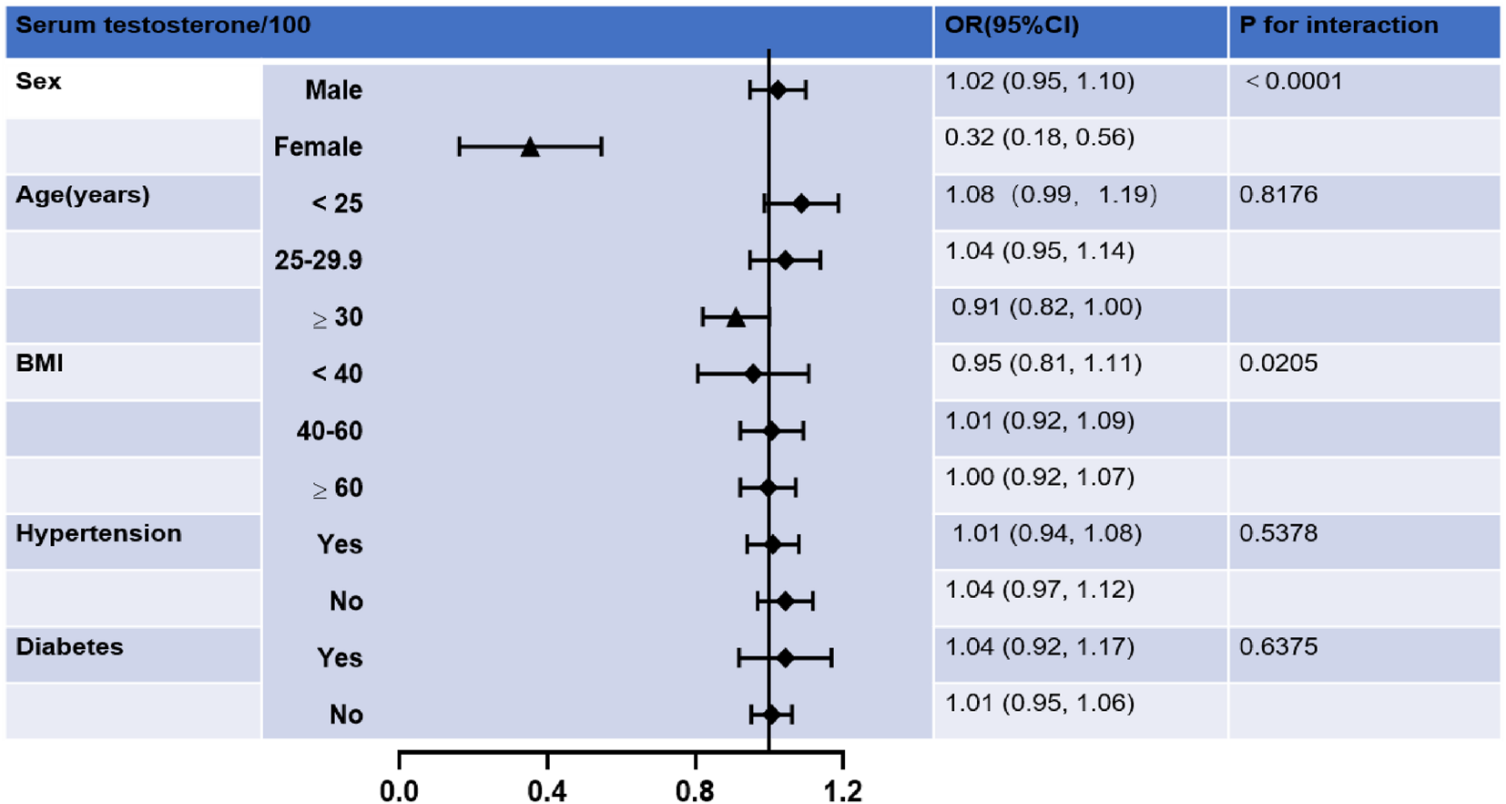
Subgroup analysis of the association between serum testosterone/100 and arthritis.

Further subgroup analysis revealed that the association of serum testosterone with arthritis was not consistent, as shown in Fig. 3. In subgroups stratified by sex, BMI, testosterone was significantly associated with arthritis (*P* < 0.05). The interaction test showed a statistically significant difference in the association between testosterone and arthritis between sex, BMI ≥ 30 kg/m^2^. In addition, age, hypertension, and diabetes were not significantly dependent on this (*P* for interaction > 0.05).

## Discussion

In our cross-sectional study, we discovered that lower serum testosterone was associated with a higher risk of arthritis. This relationship remained constant even after the addition of other factors (age, sex, race, education level, marital status, BMI, alcohol status, smoking status, hypertension, diabetes, CVD, estradiol, SHBG, income to poverty ratio,). Our dose—response analysis also showed nonlinear and negative associations between serum testosterone and the prevalence of arthritis. Additionally, we further investigated potential sex and BMI differences using subgroup analysis. This association was similar across sex as well as BMI stratification.

In this study, patients with arthritis had significantly lower serum testosterone levels than the non-arthritic population. This is consistent with several other studies showing that patients with arthritis have lower testosterone levels than the general population. Lashkari et al.^18^ demonstrated that serum testosterone levels in females with RA were lower compared with the healthy gender- and age-matched controls, higher levels of testosterone in young males may somewhat play protective roles against RA. Sowers MF et al.^19^ found that lower serum testosterone levels were associated with a higher prevalence of hand OA, but not KOA. In a larger study of 309 overweight and obese (BMI ≥ 28 kg/m^2^) adults age ≥ 60 years with KOA, higher testosterone levels were found to be associated with less Western Ontario and McMaster Universities Osteoarthritis Index (WOMAC) stiffness among men and better WOMAC function among women^20^. The relationship between serum testosterone and arthritis was at least partially correlated with the effects of sex, and BMI, as revealed by our in-depth study of the nonlinear and negative association between serum testosterone and arthritis.

**Figure 4:**
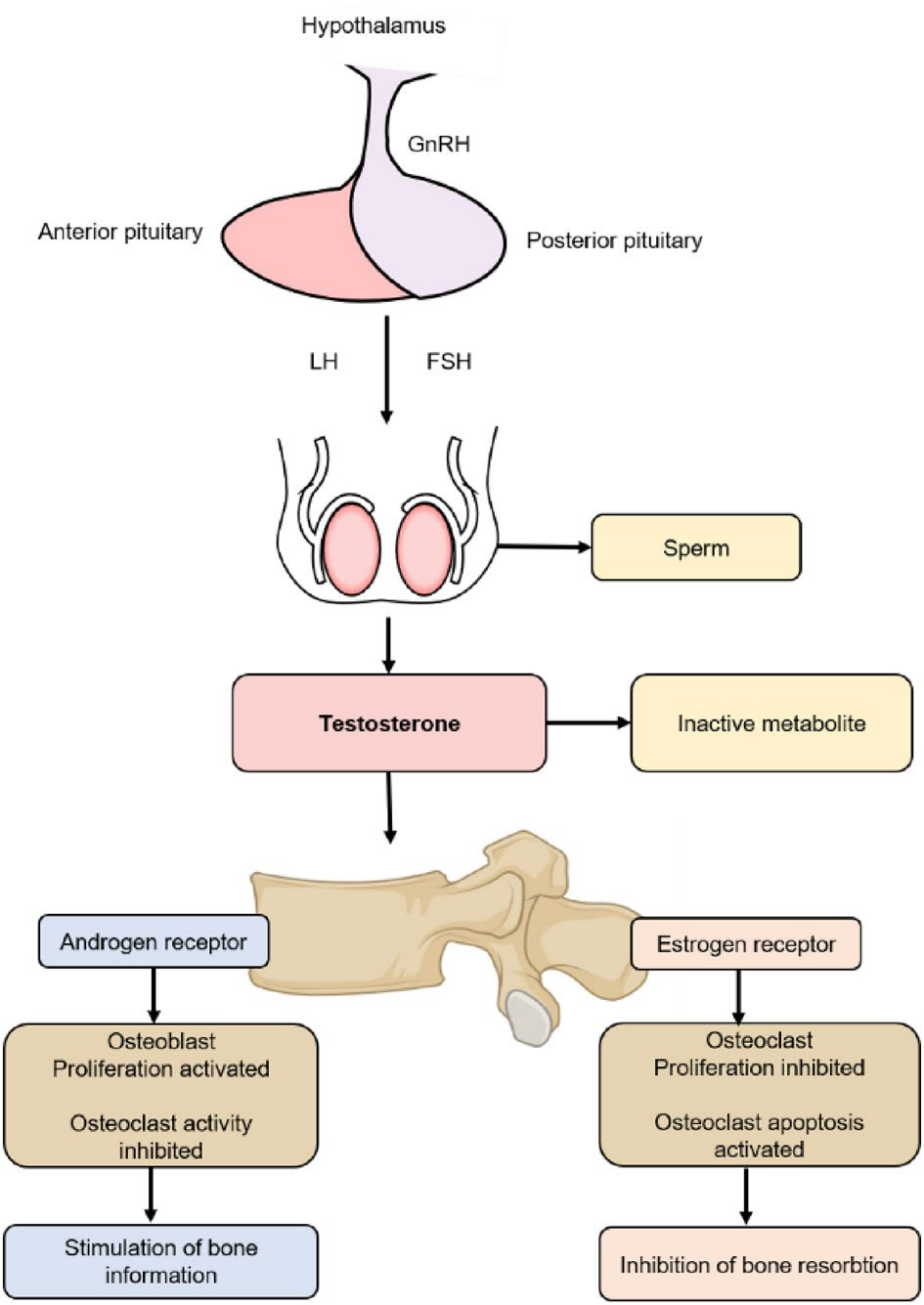
Androgens and bone. Bone metabolism is strongly regulated by androgen receptor (AR) and estrogen receptor (ER) activation. Androgens such as testosterone, after aromatization to 17β-estradiol, bind directly to AR and ER and activate them. AR activation can stimulate osteoblast proliferation and block osteoclast activity, thereby inducing bone formation. On the contrary, ER activation inhibits osteoclast proliferation and stimulates its apoptosis, thereby inhibiting bone.

In a sex-stratified analysis, we found that female arthritis patients were significantly associated with testosterone levels after adjustment. The association between sex hormones and arthritis has been widely studied based on the differences in arthritis manifested by sex differences. In particular, estradiol and testosterone have been reported to have a possible correlation with the severity of OA^21^, depletion or altered metabolism of these hormones has been considered as a risk factor for OA^22^. Androgens are currently considered to be natural immunosuppressive agents that suppress both humoral and cellular immunity and exhibit natural anti-inflammatory effects. Androgens also play an important role in keeping men less susceptible to autoimmune diseases, as the immune systems of the two sexes differ, with women having more active humoral and cellular immunity than men. It is believed that androgens can alter the immune system by acting directly on lymphocytes or on the hypothalamic-pituitary axis to regulate the levels of cytokines, immunoglobulins, and lymphocyte function^23^. Testosterone is the main androgen in the human body, and it binds to intracellular specific receptors to form active testosterone receptor complexes, which in turn bind to specific androgen response fragments (ARE) on target genes to regulate gene expression. Similar to estrogen, in addition to the direct effects of androgens on bone and cartilage, it has been shown that both estrogen and androgen receptors are present in male and female osteoblasts, and that testosterone can bind not only to androgen receptors but also to estrogen receptors to affect bone calcium metabolism homeostasis^24^. In addition, testosterone can be converted to estradiol by aromatization, which then binds to estrogen receptors and participates in the physiological regulation of bone and cartilage (Fig. 4)^25^. Decreased testosterone levels can affect cartilage metabolism through androgen receptors and ion channels, and can also cause a decrease in estradiol conversion, leading to cartilage degeneration and KOA.Furthermore, it has been controversial whether estrogen levels in humans directly mediate the pathophysiology of OA^26,27^. Sowers et al.^19^ found that higher levels of endogenous estrogen were associated with a higher incidence of knee OA, while a study by Hussain et al.^28^ showed that lower estrogen levels were a risk factor for the development of KOA. Meanwhile, a study by Hussain et al.^28^ found that high levels of SHBG were strongly associated with the development of hip OA, while Sowers et al.^19^ concluded that high levels of SHBG were not significantly associated with hand joint OA. The present study showed a negative correlation between estrogen and arthritis, while there was no significant correlation between SHBG and arthritis (Supplementary Table 1). Therefore, there is no uniform conclusion about whether endogenous estrogens are associated with the development of OA, and a large number of studies are still needed to demonstrate this in the future.

In the BMI stratified analysis, the association between serum testosterone and arthritis was significantly different in the subgroup with BMI ≥ 30 kg/m^2^. Observational studies demonstrate that obesity is associated with low serum testosterone^29^. Low testosterone may be the cause of obesity rather than its result, suggesting a potential bidirectional relationship between obesity and testosterone^30,31^. Rearranged and grown fat mass develops in men with acquired hypogonadism^32^, while in hypogonadal men, testosterone replacement could reduce fat mass^33^. In addition, obesity is a serious and direct factor that can lead to OA. The main finding of this study is that our findings support an effect of BMI on serum testosterone in association with arthritis. This causal effect may occur in the hypothalamic-pituitary–gonadal axis. Therefore, obesity as a modifiable factor, weight reduction through physical exercise and controlled diet, and lower BMI may have an important role in the relief of symptoms of OA.

In summary, regarding the effect of serum testosterone on arthritis, Newman^34^ confirmed that androgens can regulate the apoptosis of osteoblasts and osteoclasts, and that androgens promote osteoclast apoptosis while protecting osteoblasts from apoptosis, resulting in increased bone mass and bone strength. Irie et al.^35^ found increased apoptosis and reduced proliferation of chondrocytes in articular cartilage of depressed male rabbits, disorganized and irregular hierarchical arrangement of chondrocytes, and uneven coloration of the cartilage matrix. Englert et al.^36^ demonstrated that testosterone contributes to increased glycosaminoglycan content in the extracellular matrix of chondrocytes, promotes type II collagen coverage on the cartilage surface, and even affects the spanning growth of cartilage fibrous structures by in vitro culture of male calf knee cartilage. Currently, there is no unanimity of opinion in clinical practice regarding the criteria for the indication of testosterone replacement therapy for partial androgen deficiency due to aging. However, for the orthopedic field, the primary issue is to determine the association between plasma testosterone levels and androgen receptor levels in cartilage in relation to the development and progression of OA. This necessitated improvements in the means of measuring testosterone in blood to more correctly reflect the biological activity of androgens in the body, and thus the elaboration of new androgen preparations suitable for OA.

Postmenopausal women have been the focus of the majority of cohort and cross-sectional research so far. The link between serum testosterone levels and arthritis in young healthy people is poorly known. The findings of our study are extremely applicable to the entire population since we selected a nationally comprehensive sample. Furthermore, our study is based on data from NHANES and analyzed with consideration of appropriate NHANES sample weights and covariate corrections. Sensitivity analysis reduced the possibility of false positives. However, the limitations in our study cannot be ignored. First, arthritis diagnosis was based on personal interviews and recall bias was inevitable, which may lead to a risk of bias. Second, due to the cross-sectional study design, we were unable to obtain a causal relationship between testosterone and arthritis. Third, although the study used nationally representative datasets, these findings may not be universally applicable to other populations or geographical regions. In addition, since the missing data of covariables were missed randomly and the sample size was large enough to draw a conclusion, we did not use multiple imputation to deal with the missing data, thus it may influence the accuracy. Finally, residual and unmeasured confounders and measurement errors may bias our analyses. Given the limitations in the current study, these results need to be interpreted with caution and further investigations are needed to support our findings.

## Conclusion

Our studies have shown a significant association between serum testosterone levels and arthritis. The current findings emphasize the importance of serum testosterone levels in patients with arthritis. However, the findings could not establish a causal relationship and further extensive prospective studies are required.

### Supplementary Information

Supplementary Information.

## Data Availability

The survey data are publicly available on the internet for data users and researchers throughout the world (www.cdc.gov/nchs/nhanes/).

## Abbreviations

OA: Osteoarthritis
KOA: Knee osteoarthritis
BMD: Bone mineral density
NHANES: National Health and Nutrition Examination Survey
AR: Androgen receptor
CVD: Cardiovascular disease
SHBG: Sex hormone binding globulin
BMI: Body mass index
