# Supplementary material for: Lower serum testosterone is associated with increased likelihood of arthritis
Source: Sci Rep. 2023 Nov 7;13:19241. doi: 10.1038/s41598-023-46424-1 (PMC10630339; doi:10.1038/s41598-023-46424-1)
Supplement: Supplementary file 1 — Supplementary Information. [file 41598_2023_46424_MOESM1_ESM.docx]

**Supporting Information**

**Lower serum testosterone is associated with increased likelihood of arthritis**

Lulu Cheng^1,2*^ , Siyu Wang ^2^

1. College of Acupuncture-Moxibustion and Tuina, Anhui University of Chinese Medicine Hefei 230012, China
2. Graduate School, Wuhan Sports University Wuhan 430079, China

E-mail:chenglulu958@163.com

**Contents**

**1.Association between estradiol and arthritis**…………..………………………….…..3

**2. Association between SHBG and arthritis** …………………………………………....3

In the survey, we removed 5,380 testosterone, 357 estradiol, 1,195 SHBG-deficient participants, and 3,814 people with missing arthritis data from the 20,146 eligible individuals. A total of 9,400 individuals with a primary age of 20 years and older were included in this study.

**1.Association between** **estradiol and arthritis**

Table 1 shows the results of the multivariable regression analysis between estradiol /100 and arthritis. This association was significant in model 1 [0.64 (0.58,0.71)]. However, in model 2 and model 3, the negative association between estradiol and arthritis became insignificant. Sensitivity analysis using estradiol quartiles resulted in ORs of 1.00, 0.71(0.58, 0.87), 0.57(0.46, 0.72), and 0.58(0.48, 0.71) for Q1, Q2, Q3, and Q4 in model 3, respectively. Participants in the highest quartile group had a 42% reduced risk of developing arthritis compared to those in the lowest quartile of estradiol levels (P for trend < 0.05). After adjusting for all covariates, the smoothed curve fit demonstrated the nonlinear relationship between arthritis with estradiol (Figure 1).

**Table1. The association between** **estradiol and arthritis**

|  | Model1 [OR (95% CI)] | Model2 [OR (95% CI)] | Model3 [OR (95% CI)] |
| --- | --- | --- | --- |
| Estradiol/100(continuous) | 0.64(0.58,0.71) | 0.99(0.96,1.01) | 0.96(0.90,1.03) |
| Q1(2.114-12) | Reference | Reference | Reference |
| Q2(12.1-22.8) | 0.35(0.21, 0.39) | 0.84(0.71, 1.00) | 0.71(0.58, 0.87) |
| Q3(22.9-36.1) | 0.28(0.24, 0.31) | 0.74(0.61, 0.89) | 0.57(0.46, 0.72) |
| Q4(36.2-12800) | 0.20(0.17, 0.23) | 0.72(0.61, 0.85) | 0.58(0.48, 0.71) |
| P for trend | <0.0001 | 0.0005 | <0.0001 |

Model 1, no covariates were adjusted.

Model 2, age, gender, and race were adjusted.

Model 3, age, gender, race, education level, marital status, BMI, alcohol status, smoking status, hypertension, diabetes, CVD, serum testosterone, SHBG, and income to poverty ratio, were adjusted. 95% CI, 95% confidence interval; OR, odds ratio.


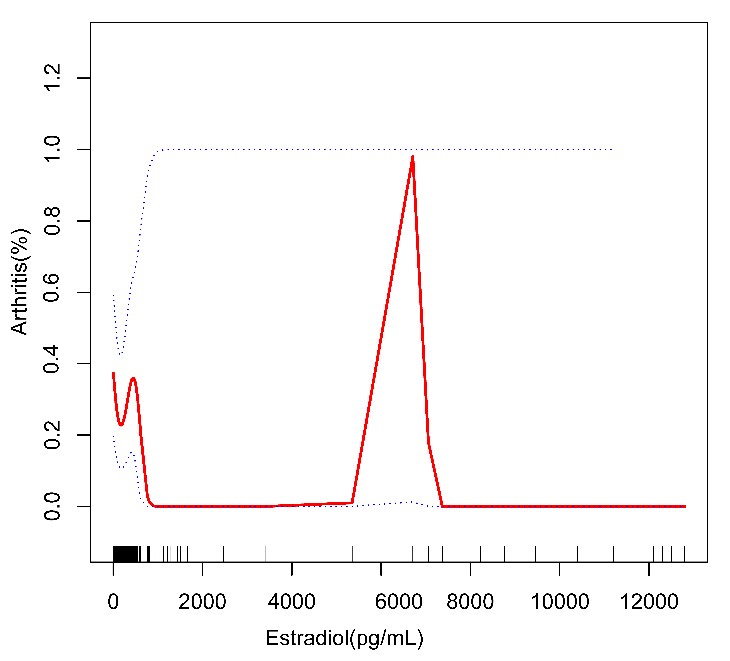


Figure1. The association between estradiol and arthritis.

**2. Association between** **SHBG and arthritis**

Table 2 shows the results of the multivariable regression analysis between SHBG/100 and arthritis. This association was significant both in model 1 [1.28(1.17,1.41)] and model 2 [0.75(0.66,0.86)]. However, in model 3, the negative association between serum testosterone and arthritis became insignificant [1.04(0.88,1.23)]. Sensitivity analyses using SHBG quartiles showed that there was no difference in the highest level quartile of SHBG in Model 3 compared with the lowest SHBG. There was no significant correlation between the SHBG and arthritis. After adjusting for all covariates, the smoothed curve fit demonstrated the nonlinear relationship between arthritis with SHBG (Figure 2).

Table2. The association between SHBG and arthritis

|  | Model1 [OR (95% CI)] | Model2 [OR (95% CI)] | Model3 [OR (95% CI)] |
| --- | --- | --- | --- |
| SHBG/100(continuous) | 1.28(1.17,1.41) | 0.75(0.66,0.86) | 1.04(0.88,1.23) |
| Q1(6.7-32.6) | Reference | Reference | Reference |
| Q2(32.61-48.13) | 1.43(1.24, 1.64) | 0.83(0.71, 0.96) | 0.96(0.80, 1.15) |
| Q3(48.14-73.56) | 1.74(1.52,2.00) | 0.72(0.61, 0.84) | 0.84(0.70, 1.02) |
| Q4(73.57-758.2) | 1.94(1.70, 2.22) | 0.64(0.54, 0.75) | 0.93(0.76, 1.14) |
| P for trend | <0.0001 | <0.0001 | 0.6324 |

Model 1, no covariates were adjusted.

Model 2, age, gender, and race were adjusted.

Model 3, age, gender, race, education level, marital status, BMI, alcohol status, smoking status, hypertension, diabetes, CVD, serum testosterone, estradiol, and income to poverty ratio, were adjusted. 95% CI, 95% confidence interval; OR, odds ratio.


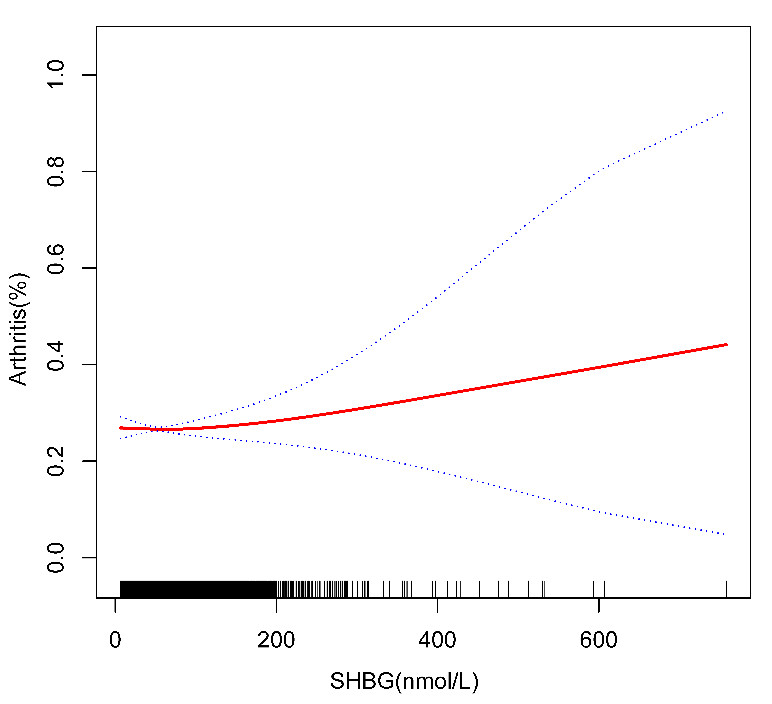


Figure2. The association between SHBG and arthritis.
